# Magnetometer-Based Drift Correction During Rest in IMU Arm Motion Tracking

**DOI:** 10.3390/s19061312

**Published:** 2019-03-15

**Authors:** Frieder Wittmann, Olivier Lambercy, Roger Gassert

**Affiliations:** Rehabilitation Engineering Lab, Department of Health Science and Technology, ETH Zurich, 8092 Zurich, Switzerland; olivier.lambercy@hest.ethz.ch (O.L.); roger.gassert@hest.ethz.ch (R.G.)

**Keywords:** wearable sensors, inertial tracking, disturbance, perturbation, virtual reality, rehabilitation, stroke, non-homogeneous, nonhomogeneous, Madgwick

## Abstract

Real-time motion capture of the human arm in the home environment has many use cases, such as video game and therapy applications. The required tracking can be based on off-the-shelf Inertial Measurement Units (IMUs) with integrated three-axis accelerometers, gyroscopes, and magnetometers. However, this usually requires a homogeneous magnetic field to correct for orientation drift, which is often not available inside buildings. In this paper, RPMC (Rest Pose Magnetometer-based drift Correction), a novel method that is robust to long term drift in environments with inhomogeneous magnetic fields, is presented. The sensor orientation is estimated by integrating the angular velocity measured by the gyroscope and correcting drift around the pitch and roll axes with the acceleration information. This commonly leads to short term drift around the gravitational axis. Here, during the calibration phase, the local magnetic field direction for each sensor, and its orientation relative to the inertial frame, are recorded in a rest pose. It is assumed that arm movements in free space are exhausting and require regular rest. A set of rules is used to detect when the user has returned to the rest pose, to then correct for the drift that has occurred with the magnetometer. Optical validations demonstrated accurate (root mean square error RMS=6.1°), low latency (61ms) tracking of the user’s wrist orientation, in real time, for a full hour of arm movements. The reduction in error relative to three alternative methods implemented for comparison was between 82.5% and 90.7% for the same movement and environment. Therefore, the proposed arm tracking method allows for the correction of orientation drift in an inhomogeneous magnetic field by exploiting the user’s need for frequent rest.

## 1. Introduction

Tracking arm movements of a user seated in front of a computer has applications in virtual reality (VR) based gaming, e.g., for the purpose of entertainment or physiotherapy [1,2,3,4]. A popular method to track arm movements relies on body worn Inertial Measurement Units (IMUs), due to the low hardware cost, low latency, and no need for a line-of-sight [5,6]. Off-the-shelf IMUs are available with integrated 3D accelerometers, gyroscopes, and magnetometers. Commonly, one IMU is fixed on each body segment of interest [7,8,9,10,11] and the orientation of the IMUs relative to the body segments is calibrated in a defined user pose, e.g., a T-pose [9]. The IMUs can then track the orientation of each body segment and joint positions can be obtained from forward kinematics [6,12]. The IMU orientation is usually obtained by integrating the angular velocity measured with the gyroscope [6,11,13,14,15,16,17]. This allows for immediate detection of orientation changes but leads to a drift of the estimated orientation over time, e.g., due to gyroscope bias [18]. Drift relative to the direction of gravity can be corrected with measurements from the accelerometer, assuming that, in the long term, gravitational acceleration dominates over translational acceleration from the subject’s movement [13,18].

However, correcting heading drift, drift around the gravitational axis, is more difficult, and a variety of solutions to this problem have been proposed. Vlasic et al. [7] used ultrasound time-of-flight to obtain an additional reference and correct for drift with a Kalman filter fusion. However, this solution requires custom hardware development, as a combination of IMU’s with ultrasound units is so far not available off-the-shelf. Other approaches, which also require custom hardware involve the use of body worn cameras [8], optical linear encoders [10], active magnetic field generators attached to the trunk [15], or permanent magnets mounted on the hand with a rigid magnetometer array attached to the trunk [19] as an additional reference source. XSens (Xsens Technologies B.V., Enschede, The Netherlands) uses a patented method, called Kinematic Coupling [20], which relies on the lateral acceleration created when walking and is hence not applicable to scenarios where the user is stationary.

Perhaps the most popular method for correcting heading drift utilizes a three-axis magnetometer to measure the surrounding magnetic field direction [11,13,16,17,19,21,22,23]. This method has the advantage that magnetometers are commonly integrated into commercial IMUs [24,25,26], which are available off-the-shelf at low cost. This approach works well for outdoor locations where the earth’s magnetic field dominates, is homogeneous, and is not parallel to gravity. However, inside buildings, the earth’s magnetic field is often heavily disturbed [27,28,29], due to, e.g., ferromagnetic structures in the walls, floor, and furniture, which make it an unreliable reference. As others have noted [27], building a complete three dimensional map of the magnetic field direction is time-consuming. More so, this map would have to be updated every time the environment changes. Several solutions have been proposed, which can tolerate a single source of magnetic disturbance for short periods of time [21,22,29]. The disturbance in the form of a change in magnetic field strength and direction relative to gravity is detected, which pauses the magnetometer-based drift correction. However, in a home scenario, sources of magnetic disturbances might be constantly in the proximity of the user, which can lead to orientation errors of more than 15° within minutes [29].

**Figure 1:**
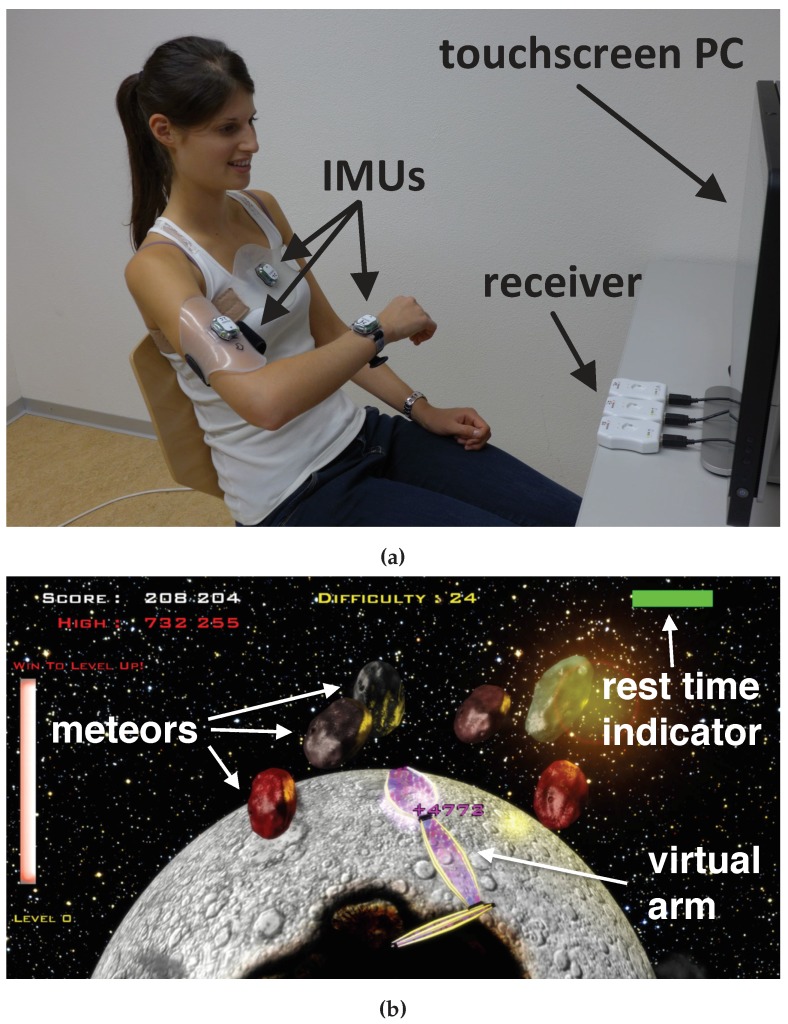
(**a**) ArmeoSenso system setup, which uses the proposed Inertial Measurement Units (IMU)-based arm tracking method (Rest Pose Magnetometer-based drift Correction (RPMC)), demonstrated by a healthy user. The receiver doubles as a battery charger for the IMUs and also guarantees the correct initial position for the calibration. (**b**) Screenshot of the Meteors therapy game [31,32], played by the experimenter during the optical validation. The purple virtual arm follows the user’s arm movement and is used to catch the meteors falling from the top of the screen. The horizontal green bar at the top right corner indicates the remaining time until the user has to assume a rest pose. Once the system recognizes the rest pose, the game is paused and the magnetometers in each sensor are used to correct accumulated drift.

This paper proposes RPMC (Rest Pose Magnetometer-based drift Correction), a method that allows inertial sensor and magnetometer-based real-time arm tracking of a user seated in front of a computer, even if the magnetic field is inhomogeneous, as one might find in a home environment. Here, the user wears three IMUs, with integrated 3-axis magnetometers, one on the trunk and one each on the upper and lower arm, which allows for real-time arm tracking, albeit with some drift around the gravitational axis. It is assumed that the user has to rest frequently between unsupported arm movements, to avoid fatigue in the arm, known as ’gorilla arm effect’ [30]. The magnetic field direction for each sensor, and its orientation, relative to the inertial frame in a specified rest pose, are recorded. The user is then asked to return to roughly the same rest pose every time he/she needs a break or after a certain amount of time has passed. A simple, rule-based classifier is used to detect whenever the user has returned to this rest pose and then the drift is corrected using the magnetometer. This tracking method is implemented in ArmeoSenso [31,32,33] (Figure 1), a VR home therapy system for upper-limb rehabilitation after neurological injury (a video demonstration of the system can be found online [34]). We present the tracking method and validate its long term accuracy and latency.

## 2. Methods

The following annotations are used. $q_{\mathrm{XY}}={\left[{q_{XY}}_{x},{q_{XY}}_{y},{q_{XY}}_{z},{q_{XY}}_{w}\right]}^{T}\in R^{4}$ is a unit-length quaternion [35] that describes the rotation from coordinate system (COS) X to Y. The symbol ⊗ indicates a quaternion multiplication and $q_{\mathrm{XY}}^{-1}=q_{\mathrm{YX}}$ is the inverse (or conjugate) of $q_{\mathrm{XY}}$.

All three dimensional vectors are extended by a 0 as their fourth value to make them compatible with quaternion multiplications. ${}_{Y}x\in R^{4}$ is a vector in coordinate system Y. $l_{\mathrm{XY}}\in R^{4}$ is a vector in coordinate system Y that points from X to Y. $\tilde{x}\in R^{4}$ is a measurement, $\bar{x}\in R^{4}$ is an average of several measurements, and $\hat{x}\in R^{4}$ is a prediction. *∂* denotes a partial derivative.

### 2.1. ArmeoSenso Therapy System

The ArmeoSenso system [31,32,33,34] is an upper limb rehabilitation platform for the home environment. It consists of a motion capture system based on the wearable sensors which are presented in this paper, a PC with a large screen for visual feedback and touch input, as well as a therapy gaming software (implemented in Unity3D, Unity Technologies Inc., San Francisco, USA). The motion capture system comprises three commercial, wireless IMUs (MotionPod 3, Movea Inc., Camarillo, USA [26]) worn on the user’s trunk, upper-arm, and wrist. Sensors are attached using ergonomic straps (Balgrist-Tec, Zurich, Switzerland). The IMUs measure acceleration ${\tilde{a}}_{i}$, angular velocity ${\tilde{\omega }}_{i}$, and the magnetic field ${\tilde{m}}_{i}$ in all three dimensions respectively, with a sampling rate of 200Hz. The range of the accelerometer is ±8.0 g and the range of the gyroscope is $\pm 2200{}^{\circ }/s$^−1^. The accelerometer and gyroscope were calibrated by the supplier (Movea Inc, USA). The magnetometer was calibrated using the accompanying Movea software in a room without magnetic interferences before the experiments. The data is streamed wirelessly at 2.4GHz to a receiver block which is connected to the PC via USB and also serves as a battery charger.

A therapy game named ’Meteors’ (Figure 1b), was implemented on ArmeoSenso, in which a virtual robot arm matches the movement of the user’s arm and is used to catch meteors falling on a planet [31,32,34]. It is used here for validating the arm tracking method. The difficulty level of the game is automatically adapted to the user’s performance in order to maintain a constant challenge and hence involves fast movements when used by a healthy user or a mildly impaired patient. Difficulty parameters are the speed of the targets and the number of targets, the interval between the creation of new targets, and the number of targets per interval. The difficulty increases based on the number of missed targets within a round of 3 min or decreases based on the remaining time before 5 targets were missed. A workspace assessment-based algorithm ensures that all reachable wrist positions are challenged during the game [31,32]. The workspace of the wrist position relative to the trunk is discretized by voxels of 10 cm side length. Targets are solely placed within the visited workspace or at neighbouring voxels. When a neighbouring voxel is reached by the wrist for the first time it is added to the workspace. In order to prevent physical exhaustion, the user is visually instructed to rest for at least 4s every 40s. A green indicator bar at the top right corner (Figure 1a) decreases in length over time and the game automatically pauses when it has vanished. Once the user assumes the rest pose, set during the calibration, the system recognizes the pose and the green indicator is filled in again. In addition, this resting phase is used for drift correction, as described in the following sections.

### 2.2. Basic Sensor Orientation Estimation

The following gyroscope-based sensor orientation estimation with a gradient descent drift correction from accelerometer and magnetometer measurements (Equations (1)–(6)), as proposed by Madgwick et al. [13], was employed. Due to its simple implementation and good performance, it serves as a base for the RPMC arm tracking algorithm proposed here. At each time step *n* of duration Δt, the estimated orientation $q_{Z_{i}S_{i}}\left[n\right]$ of each sensor frame $S_{i}$ relative to its reference frame $Z_{i}$ is updated by integrating the estimated change in orientation ${\dot{q}}_{Z_{i}S_{i}}\left[n\right]$

(1) $$q_{Z_{i}S_{i}}\left[n\right]=q_{Z_{i}S_{i}}\left[n-1\right]+{\dot{q}}_{Z_{i}S_{i}}\left[n\right]\;\cdot \;\Delta t$$

which is composed of the measured angular velocity ${\tilde{\omega }}_{i}\left[n\right]$ in the sensor frame, transposed into the reference frame $Z_{i}$, and a gradient descent based drift correction term $\mu \frac{\nabla f_{i}\left[n\right]}{||\nabla f_{i}\left[n\right]\left|\right|}$ with step-size μ

(2) $${\dot{q}}_{Z_{i}S_{i}}\left[n\right]=\frac{1}{2}q_{Z_{i}S_{i}}\left[n-1\right]⊗{\tilde{\omega }}_{i}\left[n\right]⊗q_{Z_{i}S_{i}}^{-1}\left[n-1\right]+\mu \frac{\nabla f_{i}\left[n\right]}{||\nabla f_{i}\left[n\right]\left|\right|}$$

and gradient $\nabla f_{i}\left[n\right]$.

(3) $$\nabla f_{i}\left[n\right]={\left(\frac{\partial f_{i}\left[n\right]}{\partial q_{Z_{i}S_{i}}}\right)}^{T}f_{i}\left[n\right]$$

This gradient descent serves to minimize the magnitude $\left|\right|f_{i}\left[n\right]\left|\right|\in R^{1}$ of the error function $f_{i}\left[n\right]$,

(4) $$f_{i}\left[n\right]=\left\{\begin{array}{cc} f_{g,i}\left[n\right] & \mathrm{magnetometer}\;\mathrm{not}\;\mathrm{used}\\ f_{mg,i}\left[n\right] & \mathrm{magnetometer}\;\mathrm{used} \end{array}\right.$$

which is either the difference between predicted ${}_{Z}{\hat{g}}_{i}$ and measured acceleration ${\tilde{a}}_{i}\left[n\right]$ due to gravity (transformed into the reference frame $Z_{i}$), $f_{g,i}\left[n\right]$

(5) $$f_{g,i}\left[n\right]=q_{Z_{i}S_{i}}\left[n-1\right]⊗{\tilde{a}}_{i}\left[n\right]⊗q_{Z_{i}S_{i}}^{-1}\left[n-1\right]{-}_{Z}{\hat{g}}_{i}$$

or the combined error $f_{mg,i}\left[n\right]$, which further includes the difference between the predicted ${}_{Z}{\hat{m}}_{i}\left[n\right]$ and measured magnetic field direction ${\tilde{m}}_{i}\left[n\right]$ transformed into the reference frame $Z_{i}$

(6) $$f_{mg,i}\left[n\right]=\left[\begin{array}{c} q_{Z_{i}S_{i}}\left[n-1\right]⊗{\tilde{m}}_{i}\left[n\right]⊗q_{Z_{i}S_{i}}^{-1}\left[n-1\right]{-}_{Z}{\hat{m}}_{i}\left[n\right]\\ f_{g,i}\left[n\right] \end{array}\right]$$

### 2.3. Calibration of Sensors Relative to Inertial Frame

**Figure 2:**
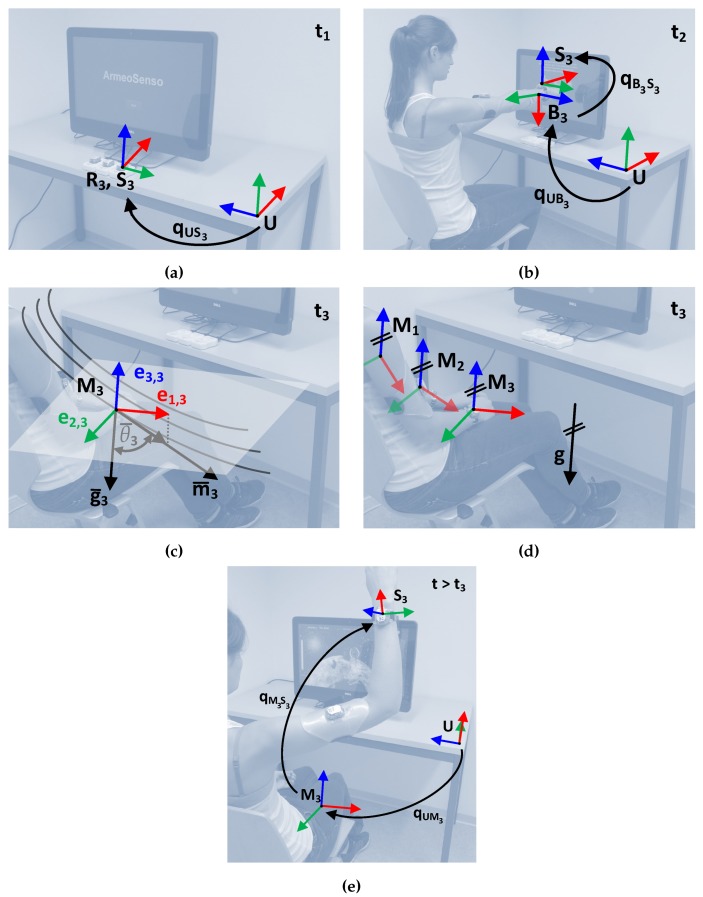
(**a**) Sensor to room calibration step ($t=t_{1}$): Relation of reference frame $R_{i}$ relative to the sensor frame $S_{i}$ and inertial frame U, here shown for the wrist sensor (i=3). (**b**) Sensor-to-body calibration step ($t=t_{2}$): Relation of body frame $B_{i}$, relative to the sensor reference frame $S_{i}$ and inertial frame U, here shown for the wrist sensor and lower arm (i=3). (**c**,**d**) Rest pose calibration step ($t=t_{3}$). (**c**) Composition of rest frame $M_{i}$ (composed of unity vectors $e_{1,3}$, $e_{2,3}$ and $e_{3,3}$) based on the gravity vector ${\bar{g}}_{i}$ measured by the accelerometer and magnetic field vector ${\bar{m}}_{i}$ measured by the magnetometer of sensor i (here wrist sensor, i=3). ${\bar{\theta }}_{3}$ is the angle between ${\bar{g}}_{3}$ and ${\bar{m}}_{3}$. Black curved lines symbolize the magnetic field lines. (**d**) Example of the relation of rest coordinate systems $M_{i}$, relative to the room for a inhomogeneous magnetic field (gravity vector g). (**e**) Tracking of the sensor frame $S_{i}$ (shown for wrist sensor i=3), relative to the rest frame $M_{i}$ and inertial frame U, after the rest pose calibration at $t_{3}$.

For the calibration, the user is first (at time $t=t_{1}$) asked to ensure that all three sensors are in the same known orientation $q_{{\mathrm{US}}_{i}}\left[n_{t_{1}}\right]$ relative to the inertial frame U, for instance in the receivers which are flat on the table and are facing towards the screen (Figure 2a). The coordinate frame U is defined with the *y*-axis parallel but in the opposite direction to gravity, the *z*-axis parallel to the PC screen, and the *x*-axis pointing towards the screen (Figure 2a). Once the orientation is confirmed by the user, by pressing a key on a keyboard placed on the desk, the constant orientation $q_{{\mathrm{UR}}_{i}}$ for all sensors can be computed with Equation (7).

(7) $$q_{{\mathrm{UR}}_{i}}=q_{{\mathrm{US}}_{i}}\left[n_{t_{1}}\right]⊗q_{R_{i}S_{i}}^{-1}\left[n_{t_{1}}\right]$$

From there on, the non-constant orientation of all sensors relative to the common inertial frame U can be computed with Equation (8).

(8) $$q_{{\mathrm{US}}_{i}}\left[n\right]=q_{{\mathrm{UR}}_{i}}⊗q_{R_{i}S_{i}}\left[n\right]\;\forall \;n_{t_{3}}>n>n_{t_{1}}$$

where $q_{R_{i}S_{i}}\left[n\right]$ is updated with Equations (1)–(5) using the reference frame $Z_{i}=R_{i}$, no magnetometer usage ($f_{i}\left[n\right]=f_{g,i}\left[n\right]$, Equation (5)), and a step-size of μ=0.03. The step-size was chosen in accordance to Madgwick et al. [13], which showed that this step size provides a good trade-off between accelerometer based drift correction and accelerometer noise rejection. This allows to track each sensors’ orientation up to the calibration of the rest pose at $t=t_{3}$. Drift around the gravitational axis that occurs during this timespan will not be corrected.

### 2.4. Calibration of Sensors Relative to Body

Next, the user has to mount the sensors on his/her body, using elastic straps. Since the exact orientation $q_{S_{i}B_{i}}$ between each sensor $S_{i}$ and the corresponding body segment COS $B_{i}$ is unknown, another calibration step is needed. The user is asked to assume a predefined pose $q_{{\mathrm{UB}}_{i}}$. We use a calibration pose in which the user’s trunk is upright and the line connecting the shoulders is parallel to the screen. The tracked arm on which the sensors are mounted is perpendicular to the user’s coronal plane ($90{}^{\circ }$ shoulder abduction and flexion) with a full elbow extension (Figure 2b). The orientation of the coordinate systems $B_{i}$ on the body are chosen according to Wu et al. [36], with the exception of using a left handed coordinate system (inverted z-axis) instead. Knowing the orientation $q_{UB_{i}}\left[n_{t_{2}}\right]$ of each body segment in the calibration pose at time $t=t_{2}$, the constant orientation $q_{S_{i}B_{i}}$ can be computed with

(9) $$q_{S_{i}B_{i}}=q_{{\mathrm{US}}_{i}}^{-1}\left[n_{t_{2}}\right]⊗q_{{\mathrm{UB}}_{i}}\left[n_{t_{2}}\right]$$

which now allows one to estimate the orientation of each body segment at all times with

(10) $$q_{{\mathrm{UB}}_{i}}\left[n\right]=q_{{\mathrm{US}}_{i}}\left[n\right]⊗q_{S_{i}B_{i}}$$

### 2.5. Position Estimation

Now, the position $p_{{\mathrm{UB}}_{i}}\left[n\right]$ of the joint frame $B_{i}$ can be estimated with forward kinematics [12]

(11) $$p_{{\mathrm{UB}}_{i}}\left[n\right]=q_{{\mathrm{UB}}_{i}}\left[n\right]⊗l_{B_{i-1}B_{i}}⊗q_{{\mathrm{UB}}_{i}}^{-1}\left[n\right]+p_{{\mathrm{UB}}_{i-1}}\left[n\right]$$

using the vector $l_{B_{i-1}B_{i}}$ that describes the constant position of one joint i−1 relative to the next joint *i*. In a similar way, the approximate position of each sensor can be estimated

(12) $$p_{{\mathrm{US}}_{i}}\left[n\right]=q_{{\mathrm{UB}}_{i}}\left[n\right]⊗l_{B_{i-1}S_{i}}⊗q_{{\mathrm{UB}}_{i}}^{-1}\left[n\right]+p_{{\mathrm{UB}}_{i-1}}\left[n\right]$$

with the position $l_{B_{i-1}S_{i}}$ of the sensor *i* relative to the previous joint. The position $p_{{\mathrm{UB}}_{0}}$ of the trunk COS relative to the room can be chosen arbitrarily, if unknown. The implemented values for $l_{B_{i-1}B_{i}}$ and $l_{B_{i-1}S_{i}}$ can be found in Table 1. If an application requires the real-world arm positions $p_{{\mathrm{UB}}_{i}}$, these values have to be adapted to the user’s body size. However, as an input method (e.g., gaming with a virtual arm) and for the rest pose classification, the positions can be treated as normalized positions, independent of the user’s body size.

### 2.6. Resting Pose Calibration and Detection

Once the sensor-to-body calibration is completed, the user is asked to assume a self-defined rest pose, e.g., trunk leaned back and hand in lap, in which the orientation of each sensor relative to its local magnetic frame $M_{i}$ is stored (Figure 2d).

First, S=100 consecutive samples (S·Δt=0.5s) from the accelerometer and magnetometer are averaged into a gravity vector ${\bar{g}}_{i}$ and the magnetic field vector ${\bar{m}}_{i}$.

(13) $${\bar{m}}_{i}=\sum_{s=0}^{S-1}\frac{{\tilde{m}}_{i}\left[n_{t_{3}}+s\right]}{S}$$

(14) $${\bar{g}}_{i}=\sum_{s=0}^{S-1}\frac{{\tilde{a}}_{i}\left[n_{t_{3}}+s\right]}{S}$$

Then, the frame $M_{i}$ for each sensor, which is spanned by ${\bar{g}}_{i}$ and ${\bar{m}}_{i}$ projected onto the plane perpendicular to ${\bar{g}}_{i}$, is computed. The orientation $q_{M_{i}S_{i}}$ of this frame relative to its corresponding sensor frame $S_{i}$ can be found by first computing a rotation matrix $R_{M_{i}S_{i}}$ and then transforming it into a quaternion. This rotation matrix consists of three unit vectors $e_{1,i}$, $e_{2,i}$, $e_{3,i}$

(15) $$R_{M_{i}S_{i}}=\left[\begin{array}{ccc} e_{1,i} & e_{2,i} & e_{3,i} \end{array}\right]=\left[\begin{array}{ccc} e_{11,i} & e_{12,i} & e_{13,i}\\ e_{21,i} & e_{22,i} & e_{23,i}\\ e_{31,i} & e_{32,i} & e_{33,i} \end{array}\right]$$

where $e_{3,i}$ is the normalized gravity vector

(16) $$e_{3,i}=\frac{{\bar{g}}_{i}}{\left|\right|{\bar{g}}_{i}\left|\right|}$$

$e_{2,i}$ is the normalized projection of the magnetic vector ${\bar{m}}_{i}$ onto the plane that is perpendicular to $e_{3,i}$ and contains the origin of the frame $M_{i}$, computed with the cross product ×

(17) $$e_{2,i}=e_{3,i}\times \frac{{\bar{m}}_{i}}{\left|\right|{\bar{m}}_{i}\left|\right|}$$

and $e_{1,i}$ is perpendicular to the plane spanned by $e_{2,i}$ and $e_{3,i}$

(18) $$e_{1,i}=e_{2,i}\times e_{3,i}$$

The quaternion $q_{M_{i}S_{i}}$

(19) $$q_{M_{i}S_{i}}={\left[q_{MS_{i},x},q_{MS_{i},y},q_{MS_{i},z},q_{MS_{i},w}\right]}^{T}$$

is then computed from the elements (Equation (15)) of the rotation matrix $R_{M_{i}S_{i}}$

(20) $$q_{MS_{i},w}=\frac{\sqrt{1+e_{11,i}+e_{22,i}+e_{33,i}}}{2}$$

(21) $$q_{MS_{i},x}=\frac{e_{32,i}-e_{23,i}}{4\;q_{MS_{i},w}}$$

(22) $$q_{MS_{i},y}=\frac{e_{13,i}-e_{31,i}}{4\;q_{MS_{i},w}}$$

(23) $$q_{MS_{i},z}=\frac{e_{21,i}-e_{12,i}}{4\;q_{MS_{i},w}}$$

A real-time estimate of $q_{{\mathrm{US}}_{i}}\left[n\right]$ with magnetic correction can be achieved by computing the constant transformation $q_{M_{i}R_{i}}$ with

(24) $$q_{M_{i}S_{i}}\left[n_{t_{3}}\right]=q_{M_{i}R_{i}}⊗q_{R_{i}S_{i}}\left[n_{t_{3}}\right]$$

(25) $$q_{M_{i}R_{i}}=q_{R_{i}S_{i}}^{-1}\left[n_{t_{3}}\right]⊗q_{M_{i}R_{i}}$$

Starting with $q_{M_{i}S_{i}}\left[n_{t_{3}}\right]$ as the initial value, $q_{M_{i}S_{i}}\left[n\right]$ is now estimated with the gradient descent algorithm Equations (1)–(4), using the reference system $Z_{i}=M_{i}$ to compute the orientation of the sensor in the room $q_{{\mathrm{US}}_{i}}\left[n\right]$ with the following equation

(26) $$q_{{\mathrm{US}}_{i}}\left[n\right]=q_{{\mathrm{UR}}_{i}}⊗q_{M_{i}R_{i}}^{-1}⊗q_{M_{i}S_{i}}\left[n\right]\;\forall \;n>n_{t_{3}}$$

which replaces the previously used Equation (8).

This concludes the calibration and the arm tracking can be started. Every 40s, the user is asked to return to the rest pose for at least 4s. Five criteria with heuristically chosen thresholds (Table 2, corresponding to the chosen kinematic values in Table 1) are used to decide whether the arm is in the rest pose. All of them have to be fulfilled for a ’rest pose detection’, which triggers the use of the magnetometer (Equation (6)). First, the measured translational acceleration (1) and angular velocity (2) for each sensor have to be close to zero (Table 2). Further, the magnetic field magnitude $\left|\right|m_{i}\left[n\right]\left|\right|$ for all I=3 sensors has to be similar to the average magnitude during calibration $\left|\right|{\bar{m}}_{i}\left|\right|$ (3) and so does the position of each sensor $p_{{\mathrm{US}}_{i}}\left[n\right]$ compared to the averaged position ${\bar{p}}_{{\mathrm{US}}_{3}}$(4). In addition, the angle $\theta_{i}\left[n\right]$ (Figure 2c) between the measured earth acceleration ${\tilde{a}}_{i}\left[n\right]$ and the magnetic field vector ${\tilde{m}}_{i}\left[n\right]$

(27) $$\theta_{i}\left[n\right]=\arccos\left(\frac{{\tilde{m}}_{i}\left[n\right]\circ {\tilde{a}}_{i}\left[n\right]}{\left|\right|{\tilde{m}}_{i}\left[n\right]||\cdot ||{\tilde{a}}_{i}\left[n\right]\left|\right|}\right)$$

should be similar to the averaged angle ${\bar{\theta }}_{i}$ (analog to Equation (13)) during calibration at time $t_{3}$.

If all these five criteria for all I=3 sensors are fulfilled, the pose is classified and the magnetometer in each sensor *i* is used to update the orientation (Equation (4)) which corrects drift around the gravitational axis that accumulated during the arm movement. At all other times, the magnetometer is not used, as the magnetic field orientation is not deemed to be reliable. To allow for a faster drift correction, the step-size μ (Equation (2)) is increased during rest.

(28) $$\mu =\left\{\begin{array}{cc} 0.1 & \mathrm{magnetometer}\;\mathrm{used}\\ 0.03 & \mathrm{magnetometer}\;\mathrm{not}\;\mathrm{used} \end{array}\right.$$

### 2.7. Alternative Methods for Comparison

Three alternative methods were implemented to compare how algorithms not relying on the frequently assumed rest pose perform for the same magnetic environment and movements. They are based on the same hardware and the Madgwick algorithm [13] but differ in when they use the magnetometer, as well as in the used magnetic reference frame $M_{i}$. The first method, denoted as ’IMU’ [13] here, never uses the magnetometer (Equation (4), step-size μ=0.03) and is hence expected to drift substantially over time. The second method, denoted as ’MARG’ [13], uses the magnetometer continuously (Equation (4), step-size μ=0.03) and records its magnetic reference frames $M_{i}$ (Equations (15)–(26)) at the same time $t_{1}$ as it performs the calibration towards the inertial frame U. The third method, denoted as ’adaptive’, mimics existing magnetic disturbance rejection methods [21,22]. This method also records the magnetic reference frames $M_{i}$ at $t_{1}$, but turns the magnetometer correction (Equation (4)) on and off depending on the magnetic field, using the same criteria for the magnetic magnitude and angle with gravity (criteria 4 and 5 in Table 2) as the proposed RPMC method, but without the position and movement criteria (criteria 1–3 in Table 2).

### 2.8. Optical Orientation Validation

**Figure 3:**
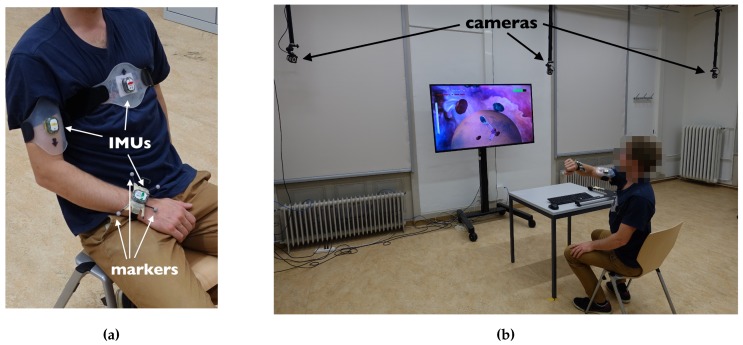
(**a**) The rest pose with IMUs mounted on the chest, upper arm, and wrist, including four retro-reflective markers for optical tracking on the wrist. (**b**) Camera and screen set up for the optical orientation validation. An experimenter plays the Meteors therapy game which uses our method for arm pose reconstruction and involves fast arm movements. The green bar at the top right corner of the screen indicates how much time is left until the user has to return to the rest pose.

The orientation estimation accuracy of the RPMC and alternative methods was validated for the wrist sensor i=3, with an optical tracking system (Optitrack S250e, Naturalpoint Inc., USA) with twelve cameras tracking retro-reflective markers at 200 Hz (Figure 3b). Four markers were fixed on the sensor mount of the wrist (Figure 3a) of a single experimenter (male, 30 years old, author FW). Informed consent was given and the experiment was conducted in accordance with the Declaration of Helsinki. The healthy subject data, used in this paper to validate the pose estimation algorithm, consisted of measurements collected in the context of a larger study that was approved by the local ethics committee (KEK-ZH: 2013–0182). A proprietary software (Motive, Naturalpoint Inc., USA) was used to track the orientation $q_{\mathrm{UO}}$ of the wrist sensor marker frame O in the inertial frame U. The definition of the inertial frame U in the optical tracking software was achieved by placing an optical calibration square (L-shaped structure with three retro-reflective markers) aligned with the table on which the sensors are placed. The orientation was streamed over UDP (MotiveToUnity v2.3, USC School of Cinematic Arts, Los Angeles, USA) to the ArmeoSenso software.

In the chosen validation setup, there were magnetic disturbances from ferromagnetic steel in the chair’s frame on which the user was seated, (Figure 3a,b, the frame of the table (Figure 3b) on which the sensors were aligned during the sensor-to-room calibration at $t_{1}$ (as well as during magnetic reference calibration for the methods ’MARG’ and ’adaptive’), and in the floor of the room. As a measure of magnetic field distortion, the normalized standard deviation of the magnetic field norm of sensor 3 $\left|\right|m_{3}\left|\right|$ for each validation session is reported.

At the time of the sensor-to-room calibration $t_{1}$, the markers, corresponding to the marker frame O, were already fixed rigidly to sensor 3. Hence, the constant relative orientation $q_{S_{3}O}$ could be computed with Equation (29).

(29) $$q_{S_{3}O}=q_{{\mathrm{US}}_{3}}^{-1}\left[n_{t_{1}}\right]⊗q_{\mathrm{UO}}\left[n_{t_{1}}\right]$$

Thus, the orientation mismatch $q_{\mathrm{UE}}\left[n\right]$ in the inertial frame U at any time $t>t_{3}$ can be computed as

(30) $$q_{\mathrm{UE}}\left[n\right]=q_{{\mathrm{US}}_{3}}\left[n\right]⊗q_{S_{3}O}⊗q_{\mathrm{UO}}^{-1}\left[n\right]=\left[\begin{array}{c} q_{UE,x}\left[n\right]\\ q_{UE,y}\left[n\right]\\ q_{UE,z}\left[n\right]\\ q_{UE,w}\left[n\right] \end{array}\right]$$

The angle between $q_{{\mathrm{UO}}_{3}}\left[n\right]$ and $q_{{\mathrm{US}}_{3}}\left[n\right]$ was defined as orientation error α[n] in degrees.

(31) $$\alpha \left[n\right]=2\arccos\left(q_{UE,w}\left[n\right]\right)\frac{180{}^{\circ}}{\pi }$$

No smoothening or other filters were applied.

N=7 validation sessions of 1h duration each were recorded. The root mean square (RMS) of the orientation error α[n] over all J= 720,000 samples was used as the main criterion to evaluate the proposed RPMC method and to compare between the three alternatives.

(32) $$RMS\left(\alpha \right)=\sqrt{\frac{1}{J}\sum_{n=1}^{J}\alpha^{2}\left[n\right]}$$

Due to the wireless transmissions, some packages (streamed at 200 Hz by the Movea MotionPod 3 sensors) were lost. For these cases the orientation was updated based on the previous received values. The number of lost data packages was counted and is reported as a percentage of the number of packages sent.

Faulty data transmission could also lead to incorrect values for acceleration $a_{i}\left[n\right]$, magnetic field $m_{i}\left[n\right]$, and angular velocities $\omega_{i}\left[n\right]$. Single incorrect values for $a_{i}\left[n\right]$ and $m_{i}\left[n\right]$ should not lead to large errors, as they provide absolute orientation information. However, large incorrect values $\omega_{i,j}\left[n\right]$ for the angular velocity $\omega_{i}\left[n\right]={\left[\omega_{i,1}\left[n\right]\;\omega_{i,2}\left[n\right]\;\omega_{i,3}\left[n\right]\right]}^{T}$ can lead to a large orientation errors within a single update step. Due to a lack of checksum in the proprietary transmission protocol, faulty packages could not be identified.

For sensor i=3, the number of ignored angular velocities are reported. In addition the maximum, median, and 99 percentile of the angular velocity measured by the gyroscope and the angular velocity derived from the orientation estimation of the proposed method and the optical tracking system are reported as a measure of the wrist movement speed. Each validation session of 1 h length was split into six time windows of 10 min each. As a measure of drift for all four implemented estimation methods, a non-parametric Friedman test of differences among repeated measures was used, to test for an increase ΔRMS(α) in orientation error RMS(α) over these six time windows.

(33) $$\Delta RMS\left(\alpha \right)=\sqrt{\frac{6}{J}\sum_{n=\frac{5/6}{J}}^{J}\alpha^{2}\left[n\right]}-\sqrt{\frac{6}{J}\sum_{n=1}^{\frac{1}{6}J}\alpha^{2}\left[n\right]}$$

To compare the total, 1 h duration, RMS orientation error RMS(α) for each of the four implemented estimation methods, a non-parametric Wilcoxon signed-rank test was used. The same test was applied at each of the six 10 min long time windows. Results were considered significant at p<0.05. All numerical results are reported as mean±standarddeviation.

### 2.9. Latency Estimation

**Figure 4:**
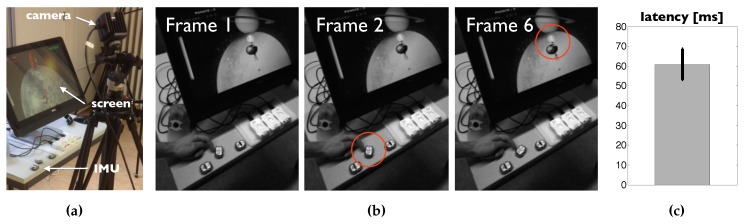
(**a**) Camera setup, screen, and IMU sensor used for latency estimation. (**b**) Example of key frames used for a single latency estimation. At Frame 1 the sensor has not yet moved, at Frame 2 the first displacement of the sensor can be visually detected, and at Frame 6 the first displacement of the virtual arm on the screen can be visually detected, resulting in an estimated latency of $6\frac{1}{120\mathrm{Hz}}=50ms$. (**c**) Mean and standard deviation of the resulting latencies (N=23).

The latency between sensor movement and rendering of the virtual arm on the PC screen (software frame rate of about 75 Hz and a screen update rate of 59 Hz) was estimated by filming the sensor and screen as a single scene (Figure 4a) with a high speed camera (OptiTrack S250e, Natural point Inc., Corvallis, USA) recording a monochrome video stream at 120 Hz. The wrist sensor was hit with a finger by the experimenter (author FW), resulting in a movement of the virtual wrist on the screen (Figure 4b). The number of frames between the onset of movement of the sensor and the onset of movement on the screen times the duration between recorded frames of the camera ($\frac{1}{120\;\mathrm{Hz}}=8.33\;ms$) results in the system’s end-to-end latency (Figure 4c). N=23 sessions were recorded and the frame differences were counted by observing the recorded video frame by frame.

## 3. Results

**Figure 5:**
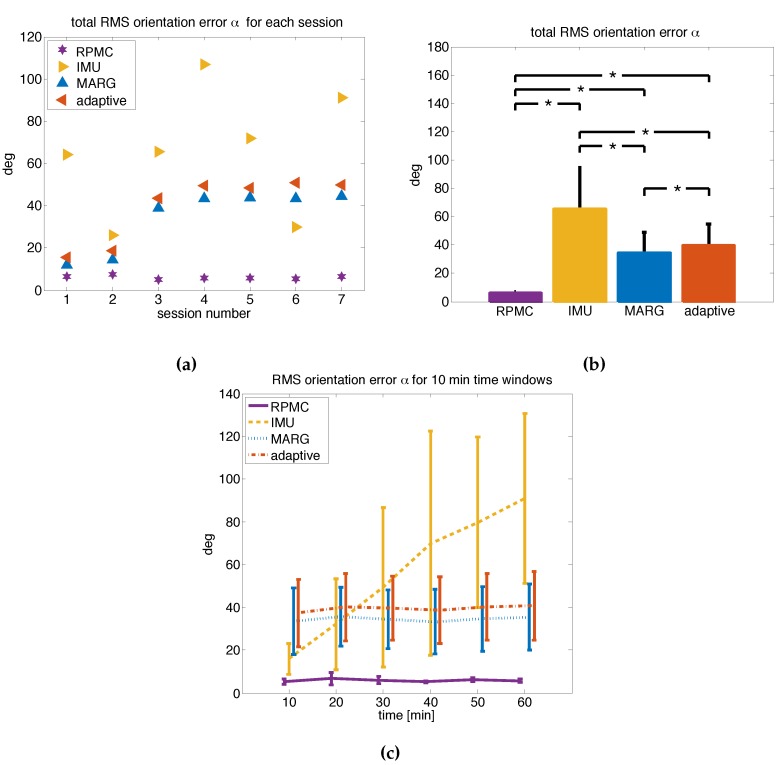
(**a**) Root mean square (RMS) of the orientation error α for each session. (**b**) Mean and standard deviation (error bars) of the RMS orientation error α in degrees of seven 1h long validation measurements. All differences are significant (* Indicates p<0.05) according to the Wilcoxon signed-rank test. (**c**) The mean and standard deviation of the RMS orientation error α for the N=7 sessions, split into six time windows of 10min each.

A summary of the results from the orientation estimation validation can be found in Table 3. For the proposed RPMC method, the average RMS error for the N=7 recorded, 1 h long sessions (Figure 5a) was $RMS\left(\alpha_{\mathrm{RPMC}}\right)=6.05\pm 0.78^{\circ }$ and significantly lower (Wilcoxon signed-rank test, p<0.05 for all three tests, Figure 5a,b) than for the three methods used for comparison with $RMS\left(\alpha_{\mathrm{IMU}}\right)=65.09\pm 29.59^{\circ }$, $RMS\left(\alpha_{\mathrm{MARG}}\right)=34.52\pm 14.59^{\circ }$ and $RMS\left(\alpha_{\mathrm{adaptive}}\right)=39.49\pm 15.40^{\circ }$. That is, the RPMC method led to an error reduction of 90.71%, 82.45%, and 84.68%, respectively.

A similar observation could be made when the recordings were split into six 10 min long windows (Figure 5c and Table 4). For each time window, the proposed RPMC method had significantly lower RMS orientation error than the other implemented methods (Wilcoxon signed-rank test, p<0.05 for all 18 tests).

There was a significant increase of the error (drift) over the six time windows for the ’IMU’ method ($\Delta RMS\left(\alpha_{\mathrm{IMU}}\right)=74.96\pm 42.82^{\circ }$, Friedman test, $\chi^{2}\left(5\right)=20.96$, p<0.01), as well as for the ’adaptive’ method ($\Delta RMS\left(\alpha_{\mathrm{adaptive}}\right)=3.28\pm 1.73^{\circ }$, Friedman test, $\chi^{2}\left(5\right)=13.86$, p<0.05), but there was no significant change over time for the proposed RPMC ($\chi^{2}\left(5\right)=5.12$, p=0.32) or the ’MARG’ method ($\chi^{2}\left(5\right)=5.86$, p=0.40).

In total, according to the classification algorithm, 20.58±2.06% of the time was spent in the rest pose, which represents a work/rest ratio of about 4:1. The magnetic field strength $\left|\right|m_{3}\left|\right|$ of the wrist sensor varied with a normalized standard deviation of 25.20±3.76% during each session. Of the J= 720,000 packages sent from sensor i=3 to the receiver block at 200Hz during each 1h long session, 1.72±1.17% were lost and an update based on the last received values was performed instead. The number of corrupt packages could not be determined.

The 99th percentile (represents 36s of each session) of the angular velocity magnitude $\left|\right|\omega_{3}\left[n\right]\left|\right|$ measured by the gyroscope of sensor i=3 was $333.1\pm 46.4{}^{\circ }s$^−1^ (mean $72.5\pm 11.0{}^{\circ }s$^−1^, median $45.6\pm 12.2{}^{\circ }s$^−1^, max $2193.9\pm 99.4{}^{\circ }s$^−1^).

The average latency between onset of sensor movement and onset of virtual arm movement on the screen was 60.9±7.7ms (Figure 4c).

## 4. Discussion

A method (RPMC) for combined inertial measurement and magnetometer-based real-time arm tracking was introduced, which corrects for drift while the user is resting, based on a pre-calibrated direction of the magnetic field. Optical validations demonstrate that this method allows for accurate (average RMS of 6.1°), low latency (61ms), fast paced (99th percentile of wrist angular velocity at $333.1{}^{\circ }s$^−1^) arm tracking for a full hour and without long term drift, despite the proximity to ferromagnetic materials as one might find in a home environment. Three alternative methods (’IMU’, ’MARG’, ’adaptive’) were implemented to compare how algorithms not relying on a frequently assumed rest pose perform for the same magnetic environment and movements. These methods showed significantly larger errors than the proposed RPMC method.

Similar to previous reports [11,18], the orientation drifted vastly ($75.0\pm 42.8{}^{\circ }h$^−1^) without any magnetometer-based correction (’IMU’ method [13]). With continuous use of the magnetometer (’MARG’ method [13]) no significant drift was observed, but the mean RMS orientation error was large ($34.5\pm 14.6{}^{\circ }$), likely due to the magnetic field disturbances caused by ferromagnetic material in the desk, chair, and floor of the validation setup, reflected in a standard deviation of magnetic field norm over the movements of 25.2%. Turning the magnetometer on and off based on magnetic measurement criteria (’adaptive’) performed significantly worse than a continuous magnetic correction (’MARG’), and even showed significant drift ($3.3\pm 1.7{}^{\circ }h$^−1^). For ease of implementation and comparison, the ’adaptive’ method was also based on the Madgwick algorithm and the thresholds for rejecting magnetic measurements were chosen identical to those of the RPMC method. However, it cannot be excluded that different thresholds would have led to better performance of the ’adaptive’ method. For the ’MARG’ and ’adaptive’ methods, the magnetic field frame calibration was performed close to a ferromagnetic structure, the steel frame of the table. This setup biases the results in favor of the proposed RPMC method. In an environment free of magnetic field disturbances, these two algorithms would likely perform better than the RPMC method. However, this would not represent the real-life situation of a home environment, where magnetic field disturbance are present, and where such technology is expected to be used.

The measured end-to-end latency (61ms) is low and in our experience prevents any perceivable mismatch between user arm movement and virtual arm movement on the screen. Optimizing the rendering process of the therapy software and increasing the sampling frequency of the gyroscope should allow us to reduce the latency further to under 50ms [18].

It should be noted that in this paper the angular velocities ${\tilde{\omega }}_{i}$ measured by the gyroscopes were streamed wirelessly to the PC before the integration was performed. Due to the high package loss (almost 2%) additional orientation drift occurred, as the most recent measured angular velocity was not available. In addition, an unknown number of corrupt packages could have led to larger drift. Lower package loss or on-board gyroscope integration, in combination with a checksum to detect faulty data packages, should lead to smaller orientation errors in the RPMC, as well as the three comparison methods. These features can be found in modern wireless IMUs (e.g., Xsens MTw Awinda). However, our results were also intended to serve as validation of the tracking performance of the ArmeoSenso system [31,32,33,34]. Hence used the older Movea MotionPod 3 sensors. The good tracking performance of the proposed algorithm despite these additional error sources further demonstrates its drift compensation abilities.

Publications on orientation filters for IMUs with magnetometer-based drift correction [11,13,22,37,38] commonly report average RMS errors, in the range of 1.7° [13] to 5.5° [11], smaller than the 6.1° reported here. In our opinion, the conditions of these experiments were carried out in favorable conditions and also less applicable to a real-world scenario like home-based arm therapy, since the validations were either carried out in environments without magnetic field disturbances [11,13,37,38] or the proximity to a single ferromagnetic source was kept brief (50s [22]).

In addition, the validations were either of short duration (1 min–5 min) [13,22,37,38] or constrained to periodic movement around a single axis, not representative of human movement [11]. As Fan et al. have recently demonstrated, even state of the art algorithms reach RMS errors of above $15{}^{\circ }$ within minutes if rotated in a challenging magnetic environment [29]. We suggest taking the relative performance of the four validated algorithms as the main result of this paper and hope that the proposed method serves future work in IMU motion tracking in challenging magnetic environments.

A number of additional in the proposed RPMC method should be considered. First, the results presented here are for a single environment using data from a single experimenter. Nevertheless, we would like emphasize that all algorithms used the same movements as input for the orientation estimation, resulting from playing the therapy game over an extended period of 7h. Different magnetic environments and movements may lead to different results, but as it is difficult to assure an environment without magnetic field distortions in most non-lab environments, our simple algorithm can be expected to perform equally well or better than the other tested algorithms, without requiring any assessment or testing in a given environment. Furthermore, experience from using the ArmeoSenso system with patients [31,32] suggested that the method works well for a wide range of environments and users. On a post-trial questionnaire 7/11 patients agreed, that “arm movements matched on screen movements” while the remaining 4/11 patients were unsure, but none disagreed with the statement, further supporting the conclusion that the proposed approach can successfully suppress drift and allow high quality motion tracking.

The drift that occurs between the room to sensor calibration and the rest pose calibration cannot be corrected and persists for the entirety of the remaining tracking session. Hence, the two calibration steps should occur in a timely manner, as implemented in the ArmeoSenso system. For this reason, a stabilization of the temperature when switching from the desk to the subject’s body is not possible, which might affect the gyroscope calibration. The acceleration, measured by the IMU, is the sum of gravity and dynamic acceleration. Dynamic acceleration reduces the pitch and roll orientation accuracy in the short term. However, the short term effect is limited, due to the small step size of μ=0.03. In the long term, periods of no dynamic acceleration (e.g., during rest), lead to correction of this error [13]. Although the magnetic field direction at each sensor position during rest can be almost arbitrary, it must not be parallel to the gravity vector. Otherwise, no information about the sensor’s heading is provided. Since the user is not required to always assume the exact same rest pose, magnetic field direction will differ slightly in a inhomogeneous field, leading to some orientation error.

It is also important that the user does not substantially change the location of the chair during a session, as this would lead to a different sensor position and magnetic field direction during rest. In the same way, if the magnetic field in the rest pose changes permanently during an ongoing session, e.g., due to the rearrangement of ferromagnetic objects, the calibration procedure has to be repeated. For such cases, the re-calibration can be automatically triggered if no rest pose has been classified despite repeated instruction to the user to assume the rest pose. This was, however, never required during the validation experiments.

The requirement for frequent rest might be seen as a limitation of the RPMC method, however in our opinion such rest is required anyway. Unsupported free form arm movements are known to lead to fatigue, also known as the ’gorilla arm effect’ [30]. While the RPMC algorithm only enforced a work/rest ratio of 10:1, the actual work/rest ratio in the experiments was about 4:1, due to voluntary rest of the experimenter during gaming as well as transition times between levels. This is still a relatively low duration of rest, compared to, e.g., work/rest ratios of 2:1 and 1:2 used in a study on fatigue in overhead work [39], which lead to reported discomfort in less than 1.5h.

To conclude, the proposed arm tracking method allows for the correction of orientation drift in an inhomogeneous magnetic field by exploiting the user’s need for frequent rest.

## Figures and Tables

**Table 1:** Implemented values for the relative position $l_{B_{i-1}B_{i}}$ between body coordinate systems $B_{i}$ and relative body to sensor positions $l_{B_{i-1}S_{i}}$ used in the forward kinematics.

Variable	Length [m]
$l_{B_{1}B_{2}}$	${\left[0,0.2,0.245\right]}^{T}$	
$l_{B_{2}B_{3}}$	${\left[0,0.26,0\right]}^{T}$	
$l_{B_{3}B_{4}}$	${\left[0,0.27,0\right]}^{T}$	
$l_{B_{1}S_{1}}$	${\left[0,0,0\right]}^{T}$	
$l_{B_{2}S_{2}}$	${\left[0,0.15,0.03\right]}^{T}$	
$l_{B_{3}S_{3}}$	${\left[0.03,0.23,0\right]}^{T}$	

**Table 2:** Criteria for classifying the rest pose. For each sensor *i* all criteria have to be satisfied for the detection of the rest pose in the proposed Rest Pose Magnetometer-based drift Correction (RPMC) method. Thresholds were chosen heuristically. Criteria No. 4 and 5 are also used for the ’adaptive’ method that is used in comparison.

No.	Description	Equation	Threshold
1	no translational acceleration	$\left|\right|a_{i}\left[n\right]\left|\right|-9.81\;m/s^{-2}$	$<0.1\;m/s^{-2}$
2	no angular velocity	$\left|\right|\omega_{i}\left[n\right]\left|\right|$	$<0.1\;\mathrm{rad}/s^{-1}$
3	similar cartesian sensor position	$\left|\right|p_{{\mathrm{US}}_{1}}\left[n\right]-{\bar{p}}_{{\mathrm{US}}_{1}}\left|\right|$	<0.2 m
		$\left|\right|p_{{\mathrm{US}}_{2}}\left[n\right]-{\bar{p}}_{{\mathrm{US}}_{2}}\left|\right|$	<0.23 m
		$\left|\right|p_{{\mathrm{US}}_{3}}\left[n\right]-{\bar{p}}_{{\mathrm{US}}_{3}}\left|\right|$	<0.25 m
4	similar magnetic magnitude	$\frac{\left|\right|m_{i}\left[n\right]-{\bar{m}}_{i}\left|\right|}{||\;{\bar{m}}_{i}\left|\right|}$	<0.3
5	similar angle of magnetic to gravity field direction	$\left|\right|\theta_{i}\left[n\right]-{\bar{\theta }}_{i}\left[n_{t_{3}}\right]\left|\right|$	<30°

**Table 3:** Summary of the validation results (RMS(α) for seven measurements of 1h each. All values are root mean square errors RMS(α) in degrees with the standard deviation following the ± symbol.

		Session						
	Total	1	2	3	4	5	6	7
RPMC	6.05±0.78	6.46	7.41	5.06	5.89	5.87	5.34	6.31
IMU	65.09±29.59	64.14	25.90	65.66	106.90	71.92	29.89	91.26
MARG	34.52±14.59	12.22	14.47	39.13	43.50	44.06	43.69	44.55
adaptive	39.49±15.40	15.67	18.83	43.38	49.51	48.44	50.87	49.68

**Table 4:** Summary of the validation results of seven measurements of 1h each, split into six, 10min long time windows. All values are root mean square errors RMS(α) in degrees with the standard deviation following the ± symbol, where applicable.

	Time Window					
	1	2	3	4	5	6
RPMC	5.34±1.25	6.75±2.99	6.02±1.69	5.24±0.42	6.22±0.94	5.76±0.80
IMU	15.90±7.07	32.21±21.23	49.40±37.23	69.95±52.51	79.74±39.80	90.86±39.73
MARG	33.48±15.55	35.47±13.76	34.32±13.72	33.32±15.18	34.61±15.11	35.34±15.43
adaptive	37.38±15.68	40.07±15.78	39.51±14.96	38.68±15.55	40.17±15.48	40.66±15.99
